# Levofloxacin versus moxifloxacin in nitazoxanide-based quadruple therapy as a first-line treatment for *Helicobacter pylori* infection: NILE, a randomized, comparative, multicenter study

**DOI:** 10.1038/s41598-026-51062-4

**Published:** 2026-05-18

**Authors:** Gasser El-Azab, Sherief Abdel Salam, Ehab Moustafa, Ahmed Abuoelhassan, Ayman Shamseya, Ahmed Ellakany, Usama Abdelaal, Medhat Assem, Fatma Khalil, Amal Mahmoud, Maha Elsabaawy, Boshra Hussein, Mohammed Ezz-Eldin, Sahar Hassany, Nariman Zaghloul, Mai Magdy

**Affiliations:** 1 Hepatology and Gastroenterology Department, National Liver Institute, Menoufia University, Shibîn El Kôm, 32511 Menoufia Egypt; 2 Tropical Medicine Department, Faculty of Medicine, Tanta University, Tanta, Egypt; 3 Tropical Medicine and Gastroenterology Department, Faculty of Medicine, Assiut University, Assiut, Egypt; 4 Gastroenterology, Hepatology and Infectious Diseases, Faculty of Medicine, Al-Azhar University, Cairo, Egypt; 5 Gastroenterology Unit, Internal Medicine Department, Faculty of Medicine, University of Alexandria, Alexandria, Egypt; 6 Gastroenterology Unit, Internal Medicine Department, Faculty of Medicine, Sohag University, Sohag, Egypt; 7 Internal Medicine Department, Faculty of Medicine, University of Bisha, Bisha, Saudi Arabia; 8 Clinical Microbiology and Immunology Department, National Liver Institute, Menoufia University, Shibîn El Kôm, Menoufia Egypt; 9 Clinical Pathology Department, Faculty of Medicine, Assiut University, Assiut, Egypt

**Keywords:** *Helicobacter pylori*, Eradication, Naïve, Resistance, Nitazoxanide, Quinolones, Levofloxacin, Moxifloxacin, Diseases, Gastroenterology, Medical research, Microbiology

## Abstract

*Helicobacter pylori* infection remains a major global health concern, with rising antibiotic resistance posing significant challenges to eradication efforts. The search for novel, effective regimens is particularly important in regions with high resistance rates to standard agents. This study evaluated the efficacy and safety of nitazoxanide-based quadruple therapies incorporating either levofloxacin or moxifloxacin. In this randomized, multicenter trial, treatment-naïve patients with confirmed *H. pylori* infection received standard triple therapy with amoxicillin, clarithromycin, and lansoprazole (ACL), levofloxacin–nitazoxanide–doxycycline–lansoprazole (LNDL) or moxifloxacin–nitazoxanide–doxycycline–lansoprazole (MNDL) for 14 days. Eradication was assessed by stool antigen testing. Rates were analyzed using intention-to-treat (ITT) and per-protocol (PP) methods. ITT eradication rates were 82.3% for the LNDL regimen, 71.8% for the MNDL regimen, and only 59.2% for the ACL regimen. Eradication with LNDL was were significantly higher than with ACL. PP analysis showed eradication rates of 87.2% for LNDL and 81.6% for MNDL, both significantly higher than ACL (60.9%). All regimens were well tolerated, with no serious adverse events reported. Fluoroquinolone-containing nitazoxanide-based quadruple regimens demonstrated superior eradication of *H. pylori* compared to clarithromycin-based triple therapy in treatment-naïve Egyptian patients. Although eradication rates did not reach the optimal threshold, these regimens represent practical alternatives when conventional regimens are not feasible.

Trial registration: ClinicalTrials.gov Identifier: NCT05184491 registered on 11-01-2022.

**Supplementary Information:**

The online version contains supplementary material available at 10.1038/s41598-026-51062-4.

## Introduction

*Helicobacter pylori* (*H. pylori*) is a common human pathogen that has been proven to be the main cause of chronic gastritis, gastric and duodenal ulcers, gastric adenocarcinoma, and gastric mucosa-associated lymphoid tissue (MALT) lymphoma^1–3^. Eradicating the pathogen helps prevent the recurrence of peptic ulcers and decreases the occurrence of gastric cancer^4,5^. However, the effectiveness of *H. pylori* therapy has markedly decreased in recent years due to the rising problem of antibiotic resistance^6,7^. The reduction in the eradication rate with standard triple therapy leads to the search for novel therapeutic regimens.

Current guidelines, including those from the American College of Gastroenterology (ACG) and the Maastricht VI/Florence Consensus, recommend bismuth-quadruple therapy as a first-line regimen^8,9^. However, implementation of these recommendations in Egypt poses significant challenges. The high prevalence of resistance to metronidazole and amoxicillin^10,11^, combined with the limited availability of tetracycline and bismuth, restricts their routine use.

Nitazoxanide (NTZ) is a broad-spectrum thiazolide antibiotic that has a spectrum of activity comparable to that of metronidazole without the drawback of acquired resistance. Published data confirm that NTZ retains activity against metronidazole-resistant strains^12–15^, making it a rational choice in a region with high metronidazole resistance.

Levofloxacin and moxifloxacin are fluoroquinolones that have demonstrated notable antibacterial effects on *H. pylori*^16^. Quinolone-based triple or quadruple regimens have been shown to be effective as first-line therapies^17–21^. Additionally, quinolone-containing therapies were found to be effective alternatives to classic regimens as a second-line approach^22–25^. The Maastricht VI/Florence Consensus recommends the use of a fluoroquinolone-containing regimen after failure of clarithromycin-based triple therapy, non-bismuth quadruple therapy, or bismuth quadruple therapy. Similarly, the ACG suggests levofloxacin-based therapy for treatment-experienced patients with persistent *H. pylori* infection, particularly when bismuth quadruple or rifabutin-based triple therapies have already been used or are unavailable^8,9^.

Although both agents belong to the same class, levofloxacin and moxifloxacin differ in key pharmacokinetic characteristics. Levofloxacin exhibits concentration-dependent bactericidal activity, near-complete oral bioavailability, and predominantly renal clearance. In contrast, moxifloxacin has a longer half-life, undergoes hepatic metabolism, and demonstrates enhanced activity against anaerobic organisms. Its relatively limited use in Egypt may be associated with lower baseline resistance.

These pharmacokinetic differences, together with the relatively low reported local resistance rates (10–20%)^10,11^, and the favorable resistance profile of NTZ, support evaluation of both agents within a nitazoxanide-based regimen in Egypt. Accordingly, we conducted the multicenter NILE trial to assess the efficacy, safety, and tolerability of levofloxacin- and moxifloxacin-containing nitazoxanide-based quadruple regimens as first-line therapy for *H. pylori* eradication.

## Methods

This prospective, multicenter, randomized, parallel-group, comparative, single-blind trial was conducted at 6 centers in Egypt: the National Liver Institute (NLI) Hospital (Menoufia University), Alexandria Main University Hospital, Tanta University Hospital, Al-Azhar University Hospital, Al-Rajhi Liver University Hospital, and Sohag University Hospital. The study adhered to Consolidated Standards of Reporting Trials (CONSORT) guidelines for reporting clinical trials.

### Study population

Consecutive patients who presented with dyspeptic symptoms for more than 1 month and underwent enzyme immunoassay *H. pylori* stool antigen tests were recruited for study participation from October 2021 to June 2024. Esophagogastroduodenoscopy (EGD) was performed for all patients older than 55 years and if clinically indicated (refractory dyspepsia, dysphagia, unintentional weight loss, or anemia).

The inclusion criteria comprised patients of either sex, aged 18–70 years, with a positive stool antigen test for *H. pylori*. Exclusion criteria included prior *H. pylori* eradication therapy, current or recent use of antibiotics (within 1 month), treatment with proton pump inhibitors, bismuth, H2 receptor antagonists, or sucralfate within 2 weeks before study entry, allergy to any of the study medications, pregnancy, active bleeding, gastric surgery, concomitant renal or hepatic impairment, or any current malignancy.

### Diagnosis of *H. pylori* infection

*H. pylori* infection status was evaluated using the *H. pylori* antigen test in the stool (Monocent, Inc., CA, USA). It is a solid-phase enzyme immunoassay based on the sandwich principle for the qualitative and quantitative detection of *H. pylori* antigen in human stool. During testing, the antigens were extracted, added to antibody-coated microwell plates supplemented with enzyme-conjugated antibodies against *H. pylori* and then incubated. If the specimens contain *H. pylori* antigens, they bind to the antibodies and simultaneously bind to the conjugate to form immobilized antibody-*H. pylori* antigen conjugate complexes. Substrates were added and then incubated to produce a blue color. The color intensity, which corresponds to the amount of *H. pylori* antigen present in the specimens, was measured with a microplate reader. A test is considered positive if the index value is greater than 1.1.

### Randomization

A computer-generated random number list was prepared by a statistician independent of the clinical investigators. Simple randomization was applied using a 2:2:1 allocation ratio (LNDL:MNDL:ACL). Sequentially numbered, sealed opaque envelopes were prepared at the NLI and opened in order of patient enrollment. Allocation was then communicated to the primary investigator at each site. Participants were blinded to their assigned regimen, while investigators were aware of treatment allocation due to differing pill counts and formulations (single-blind design).

### Treatment of *H. pylori* infection

Eligible patients who tested positive for *H. pylori* infection were randomly assigned to one of three groups. The first group received the LNDL regimen for 14 days comprising levofloxacin (750 mg OD), nitazoxanide (500 mg BD), doxycycline (100 mg BD), and lansoprazole (30 mg BD). The second group received MNDL therapy for 14 days, which included moxifloxacin (400 mg OD), nitazoxanide (500 mg BD), doxycycline (100 mg BD), and lansoprazole (30 mg BD). The third group was treated with standard triple therapy (ACL regimen) for 14 days comprising amoxicillin (1 g BD), clarithromycin (500 mg BD), and lansoprazole (30 mg BD).

### Follow-up and assessment

After completing therapy, patients were followed up in the outpatient clinic. Treatment compliance was assessed using pill counts, and any adverse effects were documented on a dedicated reporting form^26^. Compliance was defined based on the amount of medication taken, with good compliance considered as taking more than 80% of the prescribed therapy.

Four weeks after cessation of *H. pylori* treatment, a second follow-up visit was conducted. Symptom re-evaluation was performed using a questionnaire (Supplementary file 1). Additionally, reporting of any side effects and testing for *H. pylori* antigen in the stool were carried out during this visit.

### Outcomes

#### Primary outcome

The primary outcome was the successful eradication of *H. pylori* infection. Eradication success was defined as a negative *H. pylori* antigen test result after at least 4 weeks of therapy.

#### Secondary outcome

The secondary outcomes were the safety and tolerability of the study regimens. Treatment-related adverse events were assessed according to the Egyptian Pharmacovigilance Center (EPVC) criteria^26^.

### Statistical analysis

Eradication rates were calculated by both per-protocol (PP) and intention-to-treat (ITT) analyses. In the PP analysis, only eligible patients who had undergone an *H. pylori* antigen test after the completion of treatment and had taken at least 80% of the study medications were included. In the ITT analysis, all patients who had taken at least one dose of the study medication were included. It was assumed that *H. pylori* had not been eradicated if the patient failed to show up to the scheduled appointment for a stool *H. pylori* antigen test (worst-case scenario).

All eligible patients were included in the safety analyses, which compared the different treatment regimens concerning the incidence and severity of the reported clinical adverse events.

The Kolmogorov‒Smirnov test of normality revealed no significant differences in the distribution of the variables, so parametric statistics were adopted. Categorical variables were compared using the chi-square test or Fisher’s exact test, as appropriate. For multi-group comparisons with small expected frequencies, the Freeman–Halton extension of Fisher’s exact test was applied. Comparisons were carried out between the three studied normally distributed groups using ANOVA. Post hoc multiple comparisons were conducted using the Bonferroni method.

The sample size was calculated based on an anticipated 20% absolute difference in *H. pylori* eradication rates (90% vs. 70%), as reported in previous studies^12,17,27^. This corresponds to a Cohen’s effect size (h) of approximately 0.516. Using a two-tailed α of 0.05 and 80% power (1−β = 0.80), the required sample size for a two-group comparison with equal allocation was approximately 62 patients per group. To accommodate the planned 2:2:1 allocation ratio (LNDL: MNDL: ACL) and account for an anticipated 10% dropout rate, the final target enrollment was set at a minimum of 140 patients for each experimental group (LNDL and MNDL) and 70 patients for the control group (ACL), yielding a total sample size of at least 350 patients.

### Ethics

The trial adhered to the Helsinki Declaration principles and was approved by the NLI institutional review board, No. 00258/2021. All participants provided written informed consent before enrollment. This study is registered as a randomized interventional trial in Clinical Trials Registry (ClinicalTrials.gov Identifier: NCT05184491, on January 11, 2022).

## Results

### Screening and selection of participants

**Fig. 1:**
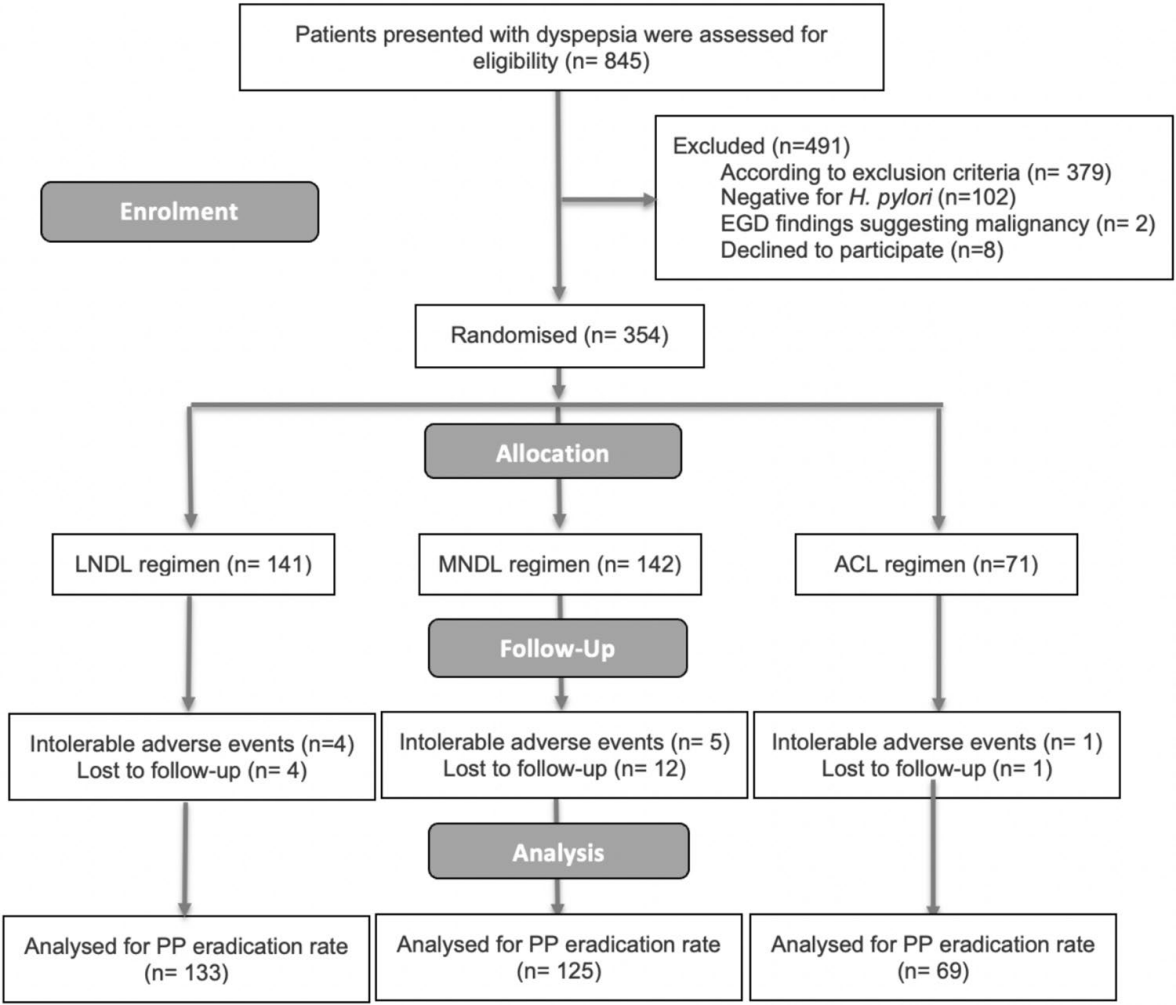
Flow chart of the study.

A total of 845 patients presenting with dyspeptic symptoms were initially evaluated. Of these, 379 patients (44.85%) were excluded based on one or more predefined exclusion criteria. The remaining 466 patients underwent testing for *H. pylori* using a stool antigen test, of whom 364 (78.11%) tested positive. Among these, 52 patients underwent EGD; 2 were subsequently excluded due to findings suggestive of gastric malignancy. Eight patients declined to participate in the study. The final study cohort therefore included 354 treatment-naïve patients. For the PP analysis, 27 patients (7.63%) were excluded: 10 due to poor compliance and 17 due to loss to follow-up (Fig. 1). Baseline demographics and symptom profiles are summarized in Table 1.

**Table 1:** Baseline clinical and demographic characteristics of the study population.

Parameter	Eradication regimen groups			*p* value
	LNDL n: 141	MNDL n: 142	ACL n: 71	
Age, mean ± SD, y	37.6 ± 12.1	37.1 ± 12.1	38.6 ± 12.2	0.7^a^
Sex, n (%)				
Female	76 (53.9%)	87 (61.3%)	43 (60.6%)	0.41^b^
Male	65 (46.1%)	55 (38.7%)	28 (39.4%)	
Symptoms, n (%)				
Epigastric pain	92 (65.2%)	81 (57%)	43 (62%)	0.95^b^
Nausea	27 (19.1%)	32 (22.5%)	15 (21.1%)	
Early satiety	34 (24.1%)	35 (24.6%)	19 (26.8%)	
Postprandial fullness	19(13.5%)	24 (16.9%)	15 (21.1%)	
Belching	13 (9.2%)	15 (10.6%)	7 (9.9%)	

^a^One-way ANOVA, ^b^Chi square test, SD: Standard deviation.

LNDL: Levofloxacin, nitazoxanide, doxycycline, lansoprazole.

MNDL: Moxifloxacin, nitazoxanide, doxycycline, lansoprazole.

ACL: Amoxicillin, clarithromycin, lansoprazole.

### Efficacy

#### Symptoms

One month after the end of treatment, the symptoms of patients who successfully eradicated *H. pylori* improved significantly more than those of patients who experienced persistent infection (82.69% vs. 55.22%, *p* < 0.001). In addition, we found no significant difference regarding symptom improvement between different treatment regimens after successful eradication.

#### Eradication

**Fig. 2:**
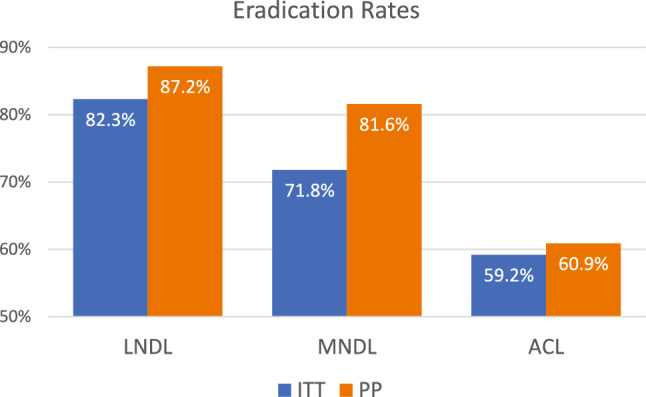
Comparative efficacy of different treatment regimens based on both intention-to-treat and per-protocol analyses.

Intention-to-treat analysis revealed eradication rates of 82.27% (116/141) for the LNDL regimen, 71.83% (102/142) for the MNDL regimen, and 59.15% (42/71) for the ACL regimen. LNDL achieved a significantly higher eradication rate than ACL (*p* < 0.001), whereas the difference between MNDL and ACL did not reach statistical significance by ITT analysis (*p* = 0.062). Direct pairwise comparison between LNDL and MNDL demonstrated a statistically significant difference by ITT analysis (*p* = 0.037). Per-protocol analysis revealed eradication rates of 87.22% (116/133) with LNDL, 81.60% (102/125) with MNDL, and 60.87% (42/69) with ACL. Both the LNDL and MNDL regimens achieved significantly higher eradication rates than ACL (*p* < 0.001 and *p* = 0.002, respectively), while the difference between LNDL and MNDL was not statistically significant by PP analysis (*p* = 0.213) (Table 2, Fig. 2).

**Table 2:** Eradication rates of *Helicobacter pylori* infection in the study population.

*H. pylori* stool antigen test result	Eradication regimen			*p* value^a^
	LNDL	MNDL	ACL	
Intention to treat	n: 141	n: 142	n: 71	0.001*
Negative (%)	116 (82.27%)	102 (71.83%)	42 (59.15%)	
95% CI	75.89–88.65	64.34–79.32	47.44–70.87	
Post hoc	*p*1 = 0.037, *p*2 < 0.001, *p*3 = 0.062			
Per-protocol	n: 133	n: 125	n: 69	< 0.001*
Negative (%)	116 (87.22%)	102 (81.6%)	42 (60.87%)	
95% CI	81.47–92.97	74.71–88.49	49.1–72.68	
Post hoc	*p*1 = 0.213, *p*2 < 0.001, *p*3 = 0.002			

^a^Chi square test, *Statistically significant, CI: Confidence interval.

*p1*: LNDL vs. MNDL; *p2*: LNDL vs. ACL; *p3*: MNDL vs. ACL.

LNDL: Levofloxacin, nitazoxanide, doxycycline, lansoprazole.

MNDL: Moxifloxacin, nitazoxanide, doxycycline, lansoprazole.

ACL: Amoxicillin, clarithromycin, lansoprazole.

### Safety and tolerance

#### Compliance

Adherence to therapy was lower in the MNDL group compared to the ACL group (88% vs. 97.2%, respectively; *p* = 0.03), with no significant differences between LNDL and ACL (*p* = 0.35) or LNDL and MNDL (*p* = 0.06). In the MNDL group, five patients discontinued treatment due to abdominal pain (n = 3), vomiting (n = 1), and dizziness (n = 1), and 12 patients were lost to follow-up. In the LNDL group, four patients discontinued therapy due to abdominal pain (n = 2), vomiting (n = 1), and severe headache (n = 1), and four patients were lost to follow-up. In the ACL group, one patient discontinued therapy due to diarrhea, and another was lost to follow-up.

No statistically significant difference was found regarding the discontinuation rate among the three study regimens (*p* = 0.85).

#### Adverse events

A total of 254 adverse events were reported in 155 patients (43.8%) among the 354 enrolled patients. The incidence of most adverse events was comparable across the three groups, with no significant differences in abdominal pain, nausea, vomiting, flatulence, constipation, dizziness, skin rash, or palpitation (all *p* > 0.05).

Diarrhea was significantly more frequent in the ACL group (16.9%) compared with the LNDL (6.38%) and MNDL (5.63%) groups (*p* = 0.011). Discolored urine was also significantly more frequent in the LNDL (11.35%) and MNDL (7.75%) groups compared with the ACL group (1.41%) (*p* = 0.041).

Headache was more common in the ACL group but did not reach statistical significance (*p* = 0.071). Adverse events led to treatment intolerance in 10 cases; however, no serious adverse events were reported (Table 3).

**Table 3:** Reported adverse events.

Adverse events	LNDL n: 141	MNDL n: 142	ACL n: 71	*p* value
Abdominal pain	14 (9.93%)	13 (9.15%)	6 (8.45%)	0.937
Nausea	23 (16.31%)	24 (16.9%)	11 (15.49%)	0.966
Vomiting	4 (2.84%)	5 (3.52%)	3 (4.23%)	0.865
Diarrhea	9 (6.38%)	8 (5.63%)	12 (16.9%)	0.011*
Constipation	7 (4.96%)	7 (4.93%)	2 (2.82%)	0.742
Flatulence	11 (7.8%)	11 (7.75%)	7 (9.86%)	0.849
Palpitation	2 (1.42%)	2 (1.41%)	1 (1.41%)	0.999
Headache	8 (5.67%)	9 (6.34%)	10 (14.08%)	0.071
Dizziness	2 (1.42%)	5 (3.52%)	2 (2.82%)	0.525
Discolored urine	16 (11.35%)	11 (7.75%)	1 (1.41%)	0.041*
Skin rash	3 (2.13%)	3 (2.11%)	2 (2.82%)	0.939

Chi square test and Fisher’s exact test were used when appropriate, *Statistically significant.

LNDL: Levofloxacin, nitazoxanide, doxycycline, lansoprazole.

MNDL: Moxifloxacin, nitazoxanide, doxycycline, lansoprazole.

ACL: Amoxicillin, clarithromycin, lansoprazole.

## Discussion

Since the early 1990s, therapeutic strategies aimed at eradicating *H. pylori* infection have progressively evolved. New treatment regimens, including triple and quadruple drug combinations, are being evaluated to achieve optimal *H. pylori* eradication rates. Eradication treatment for *H. pylori* should have at least an efficiency of 90% to be recommended for clinical practice^28,29^.

This paradigm shift in the treatment of *H. pylori* was in part stimulated by the worldwide increase in antimicrobial resistance and the associated marked decrease in cure rates with clarithromycin-based standard therapy. Because of the presence of different patterns of resistance in different populations, treatment regimens should be based on susceptibility data and should be adapted to the local resistance pattern. However, alternative strategies are being implemented to treat *H. pylori*-resistant strains, including the introduction of novel drugs, extending treatment duration, and optimizing antibiotic dosages to improve eradication regimens^30,31^.

Acid suppression is an integral part of *H. pylori* eradication therapy. In this study, we used lansoprazole, which has a chemical structure similar to that of omeprazole. However, lansoprazole is more lipophilic and therefore can penetrate the cell membrane more quickly and exhibits more potent bacteriostatic activity against *H. pylori* than omeprazole^32^. Moreover, it is the most widely available and affordable PPI in Egypt, ensuring consistent access across all six study sites.

Although clarithromycin-based triple therapy is no longer the preferred first-line regimen globally, it was selected as the control arm because it was the recommended first-line treatment for *H. pylori* infection according to Egyptian guidelines at trial initiation^33^ and was the most widely used regimen in Egypt at that time. Notably, the unsatisfactory eradication rate of 60.8% in the ACL arm by PP analysis is even lower than those reported by other studies. Basu et al. reported a cure rate of 77.6%^12^, while Raina et al. reported eradication rates of 62.8%^27^. These results further highlight the clinical need for more effective alternative regimens in this context.

The choice of antimicrobial therapy for *H. pylori* eradication depends upon the availability of drugs proven to reliably provide high cure rates locally. Only a few studies addressing *H. pylori* resistance to different antibiotics in Egyptian patients are available. Two recent studies reported that *H. pylori* exhibited high resistance to metronidazole and amoxicillin (above 80%), modest resistance to clarithromycin and azithromycin (40–52.8%), and low resistance to levofloxacin, moxifloxacin, and doxycycline (10–20%)^10,11^. These findings are not surprising since metronidazole has been overused as an over-the-counter drug in developing countries such as Egypt for various gastrointestinal infections and diarrhea, while amoxicillin and macrolides have been extensively used for the empirical treatment of respiratory tract infections in our region.

Doxycycline, a tetracycline analog, exhibits bacteriostatic activity by inhibiting bacterial protein synthesis. Current studies have reported that *H. pylori* has little resistance to this class, thus supporting its use as a superior option for first-line and rescue therapies^12,27,34^. Doxycycline was selected over tetracycline in the NILE study due to its near-complete oral bioavailability, which is minimally affected by food or divalent cations. It also offers simpler dosing, reducing pill burden and potentially improving adherence, along with better gastrointestinal tolerability. In addition, doxycycline is consistently available in Egypt, where tetracycline is often unavailable, and exhibits low resistance rates among H. pylori isolates in Egyptian patients^10,11^.

Although one observational study reported slightly higher eradication rates with tetracycline, it was nonrandomized, single-center, and included a small number of patients receiving doxycycline^35^. Additionally, its retrospective design, lack of standardized diagnostic and eradication confirmation methods, and uncertainty regarding appropriate PPI discontinuation before testing limit the generalizability of these findings.

In the NILE study, we validated the eradication rate for naïve *H. pylori* infection by using levofloxacin or moxifloxacin in nitazoxanide-based regimens. We used levofloxacin at a dosage of 750 mg/day, which is greater than that used in previous studies^12,27^. Levofloxacin exhibits concentration-dependent bactericidal activity. Increasing the dose of levofloxacin to 750 mg rises the ratio of the peak plasma concentration (C_max_) to the minimum inhibitory concentration (MIC). High levofloxacin exposure could help overcome bacterial resistance, decreasing the selection of drug-resistant mutants and improving therapeutic efficacy^36,37^. The optimal dosage of moxifloxacin for eradication therapy is unclear. Sacco et al. investigated this point and reported that 400 mg was the best dose for first-line treatment^18^.

By ITT analysis, LNDL achieved a significantly higher eradication rate than MNDL (82.27% vs. 71.83%; *p* = 0.037); however, this difference was not replicated in the PP analysis (87.22% vs. 81.60%; *p* = 0.213). This discrepancy is largely attributable to lower adherence in the MNDL group compared with the LNDL group (88% vs. 94.3%, respectively). Importantly, neither regimen achieved the ≥ 90% threshold for clinical acceptability. This suboptimal efficacy likely reflects the increasing prevalence of fluoroquinolone resistance in Egypt, driven by greater quinolone use over the past decade. Nonetheless, both LNDL and MNDL demonstrated clinically meaningful improvements over clarithromycin-based triple therapy in this Egyptian cohort, where the effectiveness of conventional regimens continues to decline.

The findings of the present NILE trial are broadly consistent with the growing body of evidence supporting NTZ-based regimens for *H. pylori* eradication^14,38^. NTZ targets pyruvate:ferredoxin/flavodoxin oxidoreductase (PFOR) through a mechanism independent of the rdxA/frxA reductive activation pathways responsible for nitroimidazole resistance, conferring a lower propensity for acquired resistance compared to metronidazole^39^.

In a meta-analysis, Iqbal et al.^13^ evaluated 13 studies comprising 1028 patients and reported a pooled eradication rate of 86% for NTZ-based regimens, with the highest subgroup rate of 92% observed in regimens combining levofloxacin, doxycycline, NTZ, and a PPI. In the Egyptian context, Hassan et al.^40^ demonstrated that adding NTZ to clarithromycin-based triple therapy as a quadruple regimen achieved ITT and PP eradication rates of 84% and 89.4%, respectively, in treatment-naïve patients. These findings parallel our LNDL results and support the use of NTZ-augmented quadruple regimens as a rational, resistance-aware strategy for *H. pylori* infection in settings characterized by high metronidazole resistance and limited availability of bismuth.

In accordance with our results, Basu et al.^12^ reported, in their multicenter randomized trial, that compared with the standard triple therapy regimen, quadruple therapy consisting of levofloxacin, omeprazole, NTZ, and doxycycline (LOAD) had a significantly greater eradication rate of *H. pylori*. The eradication rate was 93.6% for the combined LOAD (7- and 10-day courses) regimen according to PP analysis. Similarly, Rakici et al.^17^ reported that levofloxacin- and moxifloxacin-based triple regimens were more effective than standard first-line therapy (92%, 91.8%, and 82.4%, respectively, according to PP analysis), with no significant difference between levofloxacin- and moxifloxacin-based therapies. These results seem to be greater than the eradication rate achieved in our study. These differences may be attributed to differences in sample size, population type and the emergence of resistant strains due to the high consumption of quinolones in our country in the last decade^41^.

In this study, significant symptom improvement following successful *H. pylori* eradication was reported in our patients. No difference was noted between the treatment regimens used in the study. *H. pylori* eradication was found to improve dyspepsia‐associated symptoms in many studies^42–44^, and several guidelines recommend eradication therapy for *H. pylori*‐positive patients with dyspeptic symptoms to alleviate dyspeptic symptoms^23,45^.

Regarding safety and tolerability, fluoroquinolones, particularly levofloxacin, are associated with rare but potentially serious adverse effects, including tendon rupture and QT interval prolongation^46^. Although no severe adverse events were observed in our study, these considerations remain important when selecting therapy. The discontinuation rates were comparable across treatment groups. Urine discoloration occurred more frequently in the quinolone-based regimens (LNDL and MNDL) than in the clarithromycin-based regimen (ACL), likely due to the presence of nitazoxanide. Apart from diarrhea, which was significantly more common with the ACL regimen, the incidence of other adverse events was similar among all groups. These findings align with previous reports indicating an absence of major side effects for quinolone–nitazoxanide regimens^12,27,47^.

This study has several limitations. First, it was conducted using a single-blind design. Second, the absence of antibiotic susceptibility testing precluded stratification by resistance patterns and may have obscured resistance-related treatment failures; future studies should incorporate pre-treatment susceptibility data, particularly for fluoroquinolones. Third, the use of lansoprazole, a less potent proton pump inhibitor, rather than rabeprazole, esomeprazole, or potassium-competitive acid blockers, may have limited eradication efficacy; higher-potency acid suppression should be considered in future trials. Additionally, the use of clarithromycin-based triple therapy as the control arm is a notable limitation, as this regimen is no longer guideline-recommended in high-resistance settings and achieved low eradication rates. Finally, long-term follow-up was not undertaken to assess recurrence of infection.

Nevertheless, the multicenter randomized design, relatively large sample size, and inclusion of a broad geographic population across Egypt enhance the generalizability of these findings to settings with similar antimicrobial resistance patterns and limited availability of bismuth. Notably, to our knowledge, the NILE trial is the first multicenter randomized study to directly compare levofloxacin- and moxifloxacin-containing nitazoxanide-based quadruple regimens as first-line therapy for *H. pylori* eradication in a high-resistance setting.

## Conclusion

Nitazoxanide-based quadruple regimens incorporating fluoroquinolones demonstrated acceptable efficacy and safety for *Helicobacter pylori* eradication and represent a practical alternative in regions with high clarithromycin resistance and limited bismuth availability. Although eradication rates did not reach the ideal ≥ 90% threshold, levofloxacin-based therapy achieved superior results compared with classic triple therapy in treatment-naïve patients. These findings support the selective use of fluoroquinolone–nitazoxanide combinations as valuable alternatives when guideline-recommended therapies are not feasible. However, the increasing global prevalence of fluoroquinolone resistance and associated safety concerns underscore the need for antibiotic stewardship, individualized risk assessment, and large-scale trials to optimize treatment strategies in high-resistance settings.

## Supplementary Information

Supplementary Information 1.

Supplementary Information 2.

## Data Availability

The datasets used and/or analyzed during the current study are available from the corresponding author upon reasonable request.

## Abbreviations

ACL: Amoxicillin, clarithromycin, and lansoprazole
C_max_: Peak plasma concentration
EGD: Esophagogastroduodenoscopy
EPVC: Egyptian pharmacovigilance center
*H. pylori*: *Helicobacter pylori*
ITT: Intention to treat
LNDL: Levofloxacin, nitazoxanide, doxycycline, and lansoprazole
MALT: Mucosa-associated lymphoid tissue
MIC: Minimum inhibitory concentration
MNDL: Moxifloxacin, nitazoxanide, doxycycline, and lansoprazole
NLI: National liver institute
NTZ: Nitazoxanide
PFOR: Pyruvate:ferredoxin/flavodoxin oxidoreductase
PP: Per-protocol analysis
PPI: Proton pump inhibitor

## Declarations

### Trial registration

*ClinicalTrials.gov*, *NCT05184491*. Registered on January 11, 2022.
