# Supplementary material for: Levofloxacin versus moxifloxacin in nitazoxanide-based quadruple therapy as a first-line treatment for Helicobacter pylori infection: NILE, a randomized, comparative, multicenter study
Source: Sci Rep. 2026 May 18;16:15337. doi: 10.1038/s41598-026-51062-4 (PMC13184222; doi:10.1038/s41598-026-51062-4)
Supplement: Supplementary file 1 — Supplementary Information 1. [file 41598_2026_51062_MOESM1_ESM.docx]

**Project Summary**

Helicobacter pylori (H. pylori) infection is a major global health concern associated with peptic ulcer disease, chronic gastritis, and gastric cancer. The effectiveness of conventional eradication regimens has declined due to increasing antibiotic resistance. In Egypt, resistance rates to metronidazole and amoxicillin exceed 80%, while clarithromycin resistance is moderate (40–52%). Although bismuth-based quadruple therapy is recommended internationally, its availability in Egypt is limited. There is a critical need to explore alternative regimens that are effective, affordable, and feasible in this setting.

This project will evaluate two novel nitazoxanide-based quadruple regimens incorporating fluoroquinolones, Levofloxacin (LNDL) or Moxifloxacin (MNDL), compared with the standard clarithromycin-based triple therapy (ACL) in both treatment-naïve and treatment-experienced patients.

The study will be designed as a prospective, multicenter, randomized, single-blind, parallel-group clinical trial conducted at six major Egyptian hospitals. Eligible participants will be adults aged 18–70 years with confirmed H. pylori infection by stool antigen testing. After screening and informed consent, approximately 350 treatment-naïve participants will be randomized in a 2:2:1 ratio to one of the following regimens for 14 days:

LNDL: Levofloxacin, Nitazoxanide, Doxycycline, Lansoprazole

MNDL: Moxifloxacin, Nitazoxanide, Doxycycline, Lansoprazole

ACL: Amoxicillin, Clarithromycin, Lansoprazole

Treatment- experienced patients will receive either LNDL or MNDL for 14 days.

The primary objective is to compare eradication rates among the three regimens in treatment-naïve patients and between LNDL and MNDL in treatment-experienced patients. Secondary objectives include evaluating safety, tolerability, adherence, and dyspepsia symptom improvement using a structured questionnaire.

Patients will undergo a 4-week post-treatment washout, after which H. pylori eradication will be confirmed by stool antigen testing. Data will be analyzed on an intention-to-treat and per-protocol basis.

The expected outcome is to determine whether nitazoxanide-based quadruple therapy with fluoroquinolones offers superior eradication compared to conventional triple therapy and whether these regimens can be recommended as effective alternatives in Egyptian clinical practice.

**Clinical Trial Protocol**

**1. General Information**

**Title:** Levofloxacin versus moxifloxacin in nitazoxanide-based quadruple therapy as a first- and second-line treatment for Helicobacter pylori infection: NILE, a randomized, comparative, multicenter study.

**Protocol Version:** 2.0

**Date:** December 11, 2021

**Clinical Trial Registration:** ClinicalTrials.gov Identifier: NCT05184491

**Principal Investigator:** Prof. Gasser El-Azab, Hepatology and Gastroenterology Department, National Liver Institute, Menoufia University, Egypt.

**Collaborating Centers:** Tanta University, Assiut University, Al-Azhar University, Alexandria University, and Sohag University.

**Funding:** Future Pharmaceutical Industries (FPi) will provide study medications only.

**2. Background and Rationale**

Helicobacter pylori infection is a major global health issue linked to chronic gastritis, peptic ulcer disease, and gastric cancer [1-3]. Rising antibiotic resistance has reduced the effectiveness of standard therapies [4, 5]. In Egypt, resistance to metronidazole and amoxicillin exceeds 80%, while clarithromycin resistance is moderate (40-52%) [6, 7]. Bismuth-containing regimens are recommended globally but often unavailable in Egypt. Fluoroquinolone-based therapies combined with nitazoxanide (NTZ), which lacks cross-resistance with metronidazole, may provide an effective alternative. The NILE trial was designed to compare levofloxacin (LNDL) and moxifloxacin (MNDL) nitazoxanide-based quadruple regimens with standard triple therapy in treatment-naïve and experienced patients.

**3. Objectives**

**Primary Objective:**

- To compare eradication rates of LNDL, MNDL, and ACL regimens in treatment-naïve patients, and LNDL vs MNDL in treatment-experienced patients.

**Secondary Objectives:**

- To assess the safety and tolerability of these regimens.

- To evaluate compliance and improvement in dyspeptic symptoms following eradication.

**4. Study Design**

**Type:** Prospective, multicenter, randomized, single-blind, parallel-group clinical trial.

**Study Centers:** Six tertiary care hospitals across Egypt, including the National Liver Institute - Menoufia University, Tanta University, Assiut University, Al-Azhar University, Alexandria University, and Sohag University.

**Study Arms:**

*Treatment-naïve patients.*

- LNDL: Levofloxacin (750 mg OD), Nitazoxanide (500 mg BD), Doxycycline (100 mg BD), Lansoprazole (30 mg BD).

- MNDL: Moxifloxacin (400 mg OD), Nitazoxanide (500 mg BD), Doxycycline (100 mg BD), Lansoprazole (30 mg BD).

- ACL: Amoxicillin (1 g BD), Clarithromycin (500 mg BD), Lansoprazole (30 mg BD).

*Treatment- experienced patients.*

-LNDL

-MNDL

*Duration:* 14 days treatment, followed by 4-week washout before confirmation testing.

5. Study Population

**Inclusion Criteria:**

- Adults aged 18-70 years.

- Positive Helicobacter pylori stool antigen test.

- Informed consent provided.

**Exclusion Criteria:**

- Antibiotic use within the last month.

- PPI, bismuth, H2 blockers, or sucralfate within two weeks.

- Allergy to study medications.

- Pregnancy or lactation.

- Renal or hepatic impairment, prior gastric surgery, active bleeding, malignancy.

**6. Sample Size Calculation**

Based on an expected effect size (Cohen’s h = 0.516), alpha = 0.05, and power = 80%, a minimum of 62 patients per group was required. After adjusting for allocation ratio (2:2:1) and 10% dropout, the final enrollment was a minimum of 140 patients for each experimental group (LNDL and MNDL) and 70 patients for the control group (ACL).

**7. Study Procedures**

**Randomization:** Computer-generated random sequence; allocation by sealed envelopes at the coordinating center.

**Blinding:** Participants blinded to treatment; investigators aware (due to pill counts).

**Follow-up:** Pill counts for compliance; adverse events recorded. Post-treatment H. pylori stool antigen test at 4 weeks.

**8. Outcomes**

**Primary Outcome:**

- Eradication of H. pylori infection confirmed by stool antigen test ≥4 weeks after therapy.

**Secondary Outcomes:**

- Adverse events frequency and severity.

- Compliance (>80% considered good adherence).

- Dyspepsia symptom improvement.

**9. Statistical Considerations**

Analysis populations: Intention-to-treat (ITT) and per-protocol (PP).

Tests: Chi-square for proportions; ANOVA and t-tests for continuous variables; Bonferroni post hoc tests for multiple comparisons.

Significance level: p < 0.05.

Handling missing data: ITT analysis assumes treatment failure for missing data.

**10. Ethical Considerations**

The study adhered to the Declaration of Helsinki. IRB approval was obtained (National Liver Institute IRB No. 00258/2021). Written informed consent will be obtained from all participants.

**11. Data Management and Monitoring**

All data will be recorded in case report forms, anonymized, and stored securely. Adverse events will be reported according to Egyptian Pharmacovigilance Center guidelines.

**12. Publication and Dissemination Plan**

Results will be disseminated through peer-reviewed journals and scientific conferences. Individual patient data will remain confidential.

**13. References**

1. Suerbaum S, Michetti P. Helicobacter pylori infection. N Engl J Med. 2002;347:1175-86. doi:10.1056/NEJMra020542

2. Malfertheiner P, Chan FK, McColl KE. Peptic ulcer disease. Lancet. 2009;374:1449-61. doi:10.1016/S0140-6736(09)60938-7

3. Malfertheiner P. The intriguing relationship of Helicobacter pylori infection and acid secretion in peptic ulcer disease and gastric cancer. Dig Dis. 2011;29:459-64. doi:10.1159/000332213

4. Mladenova I. Epidemiology of Helicobacter pylori Resistance to Antibiotics (A Narrative Review). Antibiotics (Basel). 2023;12. doi:10.3390/antibiotics12071184

5. Megraud F, Bruyndonckx R, Coenen S, Wittkop L, Huang TD, Hoebeke M, et al. Helicobacter pylori resistance to antibiotics in Europe in 2018 and its relationship to antibiotic consumption in the community. Gut. 2021;70:1815-22. doi:10.1136/gutjnl-2021-324032

6. Metwally M, Ragab R, Abdel Hamid HS, Emara N, Elkholy H. Helicobacter pylori Antibiotic Resistance in Egypt: A Single-Center Study. Infect Drug Resist. 2022;15:5905-13. doi:10.2147/IDR.S386082

7. Asaad AM, El-Azab G, Abdelsameea E, Elbahr O, Kamal A, Abdel-Samiee M, et al. Susceptibility patterns and virulence genotypes of Helicobacter pylori affecting eradication therapy outcomes among Egyptian patients with gastroduodenal diseases. World J Gastroenterol. 2023;29:2950-60. doi:10.3748/wjg.v29.i19.2950
