# Supplementary material for: Levofloxacin versus moxifloxacin in nitazoxanide-based quadruple therapy as a first-line treatment for Helicobacter pylori infection: NILE, a randomized, comparative, multicenter study
Source: Sci Rep. 2026 May 18;16:15337. doi: 10.1038/s41598-026-51062-4 (PMC13184222; doi:10.1038/s41598-026-51062-4)
Supplement: Supplementary file 2 — Supplementary Information 2. [file 41598_2026_51062_MOESM2_ESM.pdf]

Menoufia University
National Liver Institute

Abdominal symptoms questionnaire

ID code: Date: / /

Did you expeirence during **the last week**	none	mild	moderate	severe	very severe
Pain in upper abdomen	☐	☐	☐	☐	☐
Nausea	☐	☐	☐	☐	☐
Vomiting	☐	☐	☐	☐	☐
Heartburn	☐	☐	☐	☐	☐
Regurgitation	☐	☐	☐	☐	☐
Early satiety	☐	☐	☐	☐	☐
Postprandial fullness	☐	☐	☐	☐	☐
Belching	☐	☐	☐	☐	☐
Bloating	☐	☐	☐	☐	☐
Rumbling	☐	☐	☐	☐	☐
Diffeculty in swallonig	☐	☐	☐	☐	☐
Vomiting of blood	☐	☐	☐	☐	☐
Black, tarry stool	☐	☐	☐	☐	☐
Diarrhea	☐	☐	☐	☐	☐
Constipation	☐	☐	☐	☐	☐
